# Segregation of the membrane cargoes, BACE1 and amyloid precursor protein (APP) throughout the Golgi apparatus

**DOI:** 10.1111/tra.12831

**Published:** 2022-02-13

**Authors:** Lou Fourriere, Ellie Hyun‐Jung Cho, Paul A. Gleeson

**Affiliations:** ^1^ The Department of Biochemistry and Pharmacology Bio21 Molecular Science and Biotechnology Institute, The University of Melbourne Melbourne Victoria; ^2^ Biological Optical Microscopy Platform The University of Melbourne Melbourne Victoria Australia

**Keywords:** Airyscan microscopy, amyloid precursor protein (APP), beta‐secretase 1 (BACE1), colocalization, segregation, *trans*‐Golgi network (TGN)

## Abstract

The intracellular trafficking of β‐site amyloid precursor protein (APP) cleaving enzyme (BACE1) and APP regulates amyloid‐β production. Our previous work demonstrated that newly synthesized BACE1 and APP are segregated into distinct trafficking pathways from the *trans*‐Golgi network (TGN), and that alterations in their trafficking lead to an increase in Aβ production in non‐neuronal and neuronal cells. However, it is not known whether BACE1 and APP are transported through the Golgi stacks together and sorted at the TGN or segregated prior to arrival at the TGN. To address this question, we have used high‐resolution Airyscan technology followed by Huygens deconvolution to quantify the overlap of BACE1 and APP in Golgi subcompartments in HeLa cells and primary neurons. Here, we show that APP and BACE1 are segregated, on exit from the endoplasmic reticulum and in the *cis*‐Golgi and throughout the Golgi stack. In contrast, the transferrin receptor, which exits the TGN in AP‐1 mediated transport carriers as for BACE1, colocalizes with BACE1, but not APP, throughout the Golgi stack. The segregation of APP and BACE1 is independent of the Golgi ribbon structure and the cytoplasmic domain of the cargo. Overall, our findings reveal the segregation of different membrane cargoes early in the secretory pathway, a finding relevant to the regulation of APP processing events.

## Graphical abstract

Amyloid precursor protein (APP) and the β‐secretase, BACE1, are sorted into different post‐Golgi transport pathways. Here, we investigated the partitioning of APP and BACE1 along the secretory pathway. Using super‐resolution microscopy, we show that APP and BACE1 are segregated in the *cis*‐Golgi and throughout the Golgi stack in HeLa cells and primary neurons, whereas, the transferrin receptor, colocalizes with BACE1 but not APP. These findings reveal segregation of membrane cargoes early in the secretory pathway, which we propose limits APP processing.

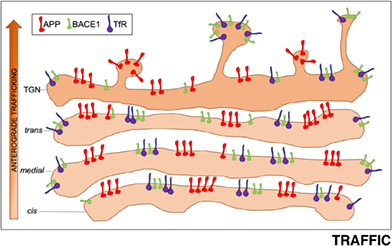

## 1 INTRODUCTION

Alzheimer's disease (AD) is a chronic and progressive neurodegenerative disease associated with senile plaques and neurofibrillary tangles in the brain and represents the most common form of dementia.^1^ Amyloid precursor protein (APP) and amyloid‐β (Aβ) peptides are central to the generation of senile plaques in AD.^2^ The main isoform of APP in neurons is a 695 amino acid type I transmembrane protein with a long N‐terminus luminal domain, a single transmembrane domain, and a short cytoplasmic tail. Aβ is produced after sequential cleavage of APP by β‐ and γ‐secretases in the endo/lysosomal and secretory pathways.^3^, ^4^, ^5^ Once produced, Aβ is secreted from the cell and aggregates to form neurotoxic oligomers and eventually senile plaques. Familial AD is associated with mutations in APP and its secretases, resulting in elevated levels of APP processing and Aβ production. The early and late endosomes are one of the major sites for intracellular APP processing^6^, ^7^, ^8^ and AD sporadic risk factors include genes of the endocytic machinery. In addition, the secretory pathway and the Golgi apparatus have recently re‐emerged as another major site for APP processing and Aβ production.^9^, ^10^, ^11^, ^12^

The β‐secretase, β‐site amyloid precursor protein cleaving enzyme (BACE1), is responsible for the rate limiting step in the cleavage of APP to generate C99 which is then further processed by γ‐secretase to Aβ.^13^, ^14^, ^15^ BACE1 is a membrane‐bound type I transmembrane aspartyl protease, synthesized as a precursor protein in the endoplasmic reticulum (ER) and which then undergoes post‐translational modifications (glycosylation, phosphorylation and furin cleavage) to be converted into a 75 kDa mature active protein.^16^, ^17^, ^18^ The optimal BACE1 activity requires an acidic environment.^19^, ^20^ The highest activity of BACE1 is detected in endosomes and in the *trans*‐Golgi network (TGN) at steady state.^13^, ^21^, ^22^

APP and BACE1 have distinct intracellular itineraries and membrane trafficking is central to their convergence at specific locations and the regulation of APP processing.^23^ For example, modification of trafficking kinetics or itinerary of BACE1 influences the regulation of APP processing and the production of Aβ^24^ and accumulation of APP in the TGN promotes Aβ production.^25^ While there have been extensive studies analysing APP transport and processing in the endocytic pathway, less is known about the mechanism(s) for regulating APP processing along the secretory pathway. An important question is how APP is protected from processing by the secretases along the same secretory pathway? Notably, recent studies by our laboratory and others have shown that BACE1 and APP are shunted into distinct post‐Golgi transport pathways at the TGN in various cell types, including neurons and the segregation of BACE1 from APP into different transport carriers at the TGN provides a mechanism for shielding APP from BACE1 activity.^9^, ^11^, ^12^

Whereas BACE1 is transported directly from the TGN to the plasma membrane (PM), the majority of APP is transported from the TGN to the early endosomes. The shunting of BACE1 and APP into different pathways is mediated by cytoplasmic sorting signals. TGN export of BACE1 is regulated by a DDISLL motif on the cytoplasmic tail which is recognized by the adaptor protein AP‐1 and ARF1 and ARF4 small Golgi proteins.^12^ Instead, the TGN export of APP is regulated by a YKFFEE sequence within its cytoplasmic tail which is recognized by AP‐4/Arl5b.^9^, ^25^ Sorting of APP and BACE1 into different post‐Golgi pathways raises the question where APP and BACE1 are segregated within the ER/Golgi transport system: is it during the sorting process in the TGN or prior to recruitment into distinct AP‐mediated transport carriers? This is a relevant question given the co‐transport of newly synthesized APP and BACE1 along the anterograde pathway and reports that retromer‐mediated recycling of APP to the TGN is required for efficient Aβ production APP.^26^ The possibility that APP and BACE1 partitioning may occur prior to recruitment into TGN‐generated transport carriers is worthy of investigation as recent findings have suggested that other membrane cargoes can be segregated from each other prior to arrival at the TGN.^27^, ^28^, ^29^, ^30^ Here, we have sought to define the level of colocalization and segregation of APP and BACE1 throughout the Golgi stack and TGN in HeLa cells and primary mouse neurons.

To perform this study, we have optimized super‐resolution (SR) microscopy and quantitative analysis to obtain high‐resolution imaging of BACE1 and APP within distinct regions of the Golgi and to determine their level of segregation. Imaging using an Airyscan detector coupled with Huygens deconvolution process obtained a lateral resolution of ~90 nm. From these analyses, we demonstrated that the two cargoes, APP and BACE1, are segregated early in the anterograde pathway of HeLa cells and primary neurons and we have examined features of these membrane proteins and Golgi organization that may contribute to this partitioning of cargoes.

## 2 RESULTS

BACE1 and APP share the same anterograde/secretory pathway through the ER and Golgi apparatus. From our previous work and studies from other laboratories, BACE1 and APP have been shown to use different adaptor proteins to exit the TGN. However, it is not known if BACE1 and APP are segregated only once they reach the TGN or whether they are spatially segregated through their entire Golgi journey. To define the specific localisation of BACE1 and APP within the Golgi stack, we used SR techniques and developed a novel method for quantitative analysis.

### 2.1 Methods for the quantification of colocalization using SR microscopy

**FIGURE 1:**
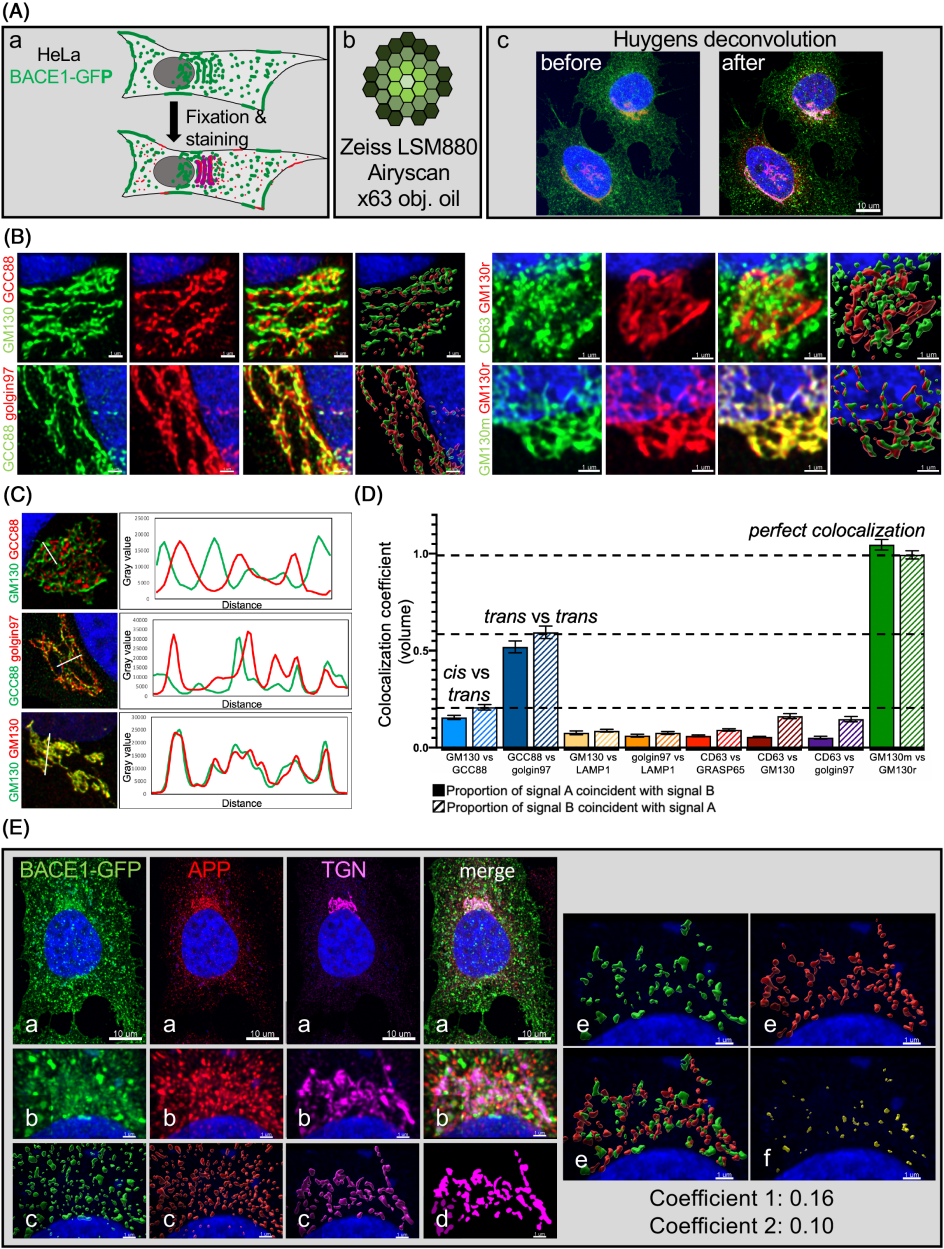
Airyscan technology to map Golgi compartments and define colocalization coefficients. (A) Outline of the experimental plan. (a) Monolayer of HeLa cells was fixed, permeabilized and blocked and then stained for different markers. (b) Diagram of concentrically arranged hexagonal detector array for Airyscan. Images were taken using a Zeiss LSM880 Airyscan with ×63 oil objective in SR mode. (c) Z‐stacks images were deconvolved using Huygens software (conservative deconvolution mode). (B and C) Monolayers of HeLa WT cells were fixed, permeabilized and blocked and then double‐stained with anti‐GM130 (green) and anti‐GCC88 (red), anti‐GCC88 (green) and anti‐golgin97 (red), anti‐CD63 (green) and anti‐GM130 (red), anti‐GM130 mouse (green) and anti‐GM130 rabbit (red) and DAPI (blue). Images from each staining pair is shown as Green, Red, Merged and 3D‐reconstructed. Bar represents 1 μm. (C) Linescans were obtained using FIJI software.^54^ (D) Colocalization coefficient (volume) analysed for different Golgi markers and organelles markers using our experimental plan. Colocalization was analysed using Imaris. The colocalization coefficient (volume) corresponds to the volume of the signal of marker A coincident with the volume of the signal marker B (= colocalization A and B) over the total volume of the signal of marker A. *cis* (GM130) versus *trans* (GCC88) colocalization coefficient is 0.21; *trans* (GCC88) versus *trans* (golgin97) colocalization coefficient is 0.59. GM130 (mouse antibody to GM130 and AF488) versus GM130 (rabbit antibody to GM130 and AF562) colocalization coefficient is 0.99. Data are represented as the mean ± SEM of three independent experiments (27 < *n* < 37). (E) Outline of BACE1 and APP colocalization measurement. (a) Monolayer of HeLa cells stably expressing BACE1‐GFP was fixed, permeabilized and blocked. Cells were then stained with anti‐APP (red), anti‐TGN (mix of GCC88 and golgin97; purple) and DAPI ddeconvolved using Huygens and analysed using Imaris. (b) Zoom in the Golgi area of BACE1‐GFP positive cells. (c) 3D‐reconstructed signal on Imaris for each channel. (d) TGN signal (purple) is used to determine the Golgi mask. (e) Signal of BACE1‐GFP (green) and APP (red) present in the TGN mask was selected. (f) Colocalization between BACE1‐GFP (green) and APP (red) inside the TGN mask was calculated (in yellow). For this cell, the colocalization coefficient of BACE1 over APP is 0.16, and the colocalization coefficient of APP over BACE1 is 0.10. Scale bars represent 10 μm or 1 μm, as indicated. APP, amyloid precursor protein; BACE1, β‐site amyloid precursor protein cleaving enzyme

In this study, HeLa cells and primary mouse neurons were used to quantify the colocalization of APP and its protease BACE1. In most of our experiments, we took advantage of stable BACE1‐GFP HeLa cells we have previously characterized and shown that the overexpression and the GFP tag of BACE1‐GFP did not affect BACE1 intracellular localization.^12^ Cells were fixed and stained for APP using a number of different APP antibody specificities and for Golgi markers (Figure 1Aa). We used a range of Golgi markers to stain the *cis* (GM130), *cis* and *medial* (Giantin), *medial* (GnT1) and *trans* Golgi (GCC88, golgin97, p230/golgin‐245) cisternae. Images were acquired using a Zeiss Airyscan microscope with an ×63 oil objective (Figure 1Ab). For each position, we took z‐stack images to cover the entire cell volume. Images were processed using Huygens deconvolution (conservative mode; Figure 1Ac). Airyscan technology is reported to provide a lateral resolution of 120 nm for 2D and 3D data and together with Huygens deconvolution, a resolution of ~90 nm (https://svi.nl/), a resolution which is sufficient to distinguish Golgi subregions, based on the ultrastructure of this organelle.^31^ We directly evaluated the resolution using 100 nm fluorescent beads (Table 1) as described in methods. The lateral resolution measured using yellow‐green fluorescent beads with 488 nm excitations was 149 nm when Zen Processed (Airyscan) and 105 nm when Huygens Processed (*n* = 12). The lowest resolution we measured was 93 nm when blue fluorescent beads was imaged with 405 nm excitation and processed with Huygens (Table 1). Therefore, we processed the images throughout this study using Huygens deconvolution. Imaris software was used to perform the quantification using the surface‐surface colocalization module (i.e., Figure 1B). Shown are the individual green and red channels after deconvolution, followed by the merge of the green and red channels, and, finally the combination of the 3D reconstructed objects of the two channels from the Imaris analysis. Images show *cis*‐Golgi (GM130) and TGN (GCC88) markers (LHS, upper panel), two TGN (GCC88 and golgin97) markers (LHS, lower panel), late endosome (CD63) and *cis*‐Golgi (GM130) markers (RHS, upper panel), and two different GM130 primary antibodies (RHS, lower panel; Figure 1B).

**TABLE 1:** Evaluation of the lateral resolution of Airyscan processed images

Excitation (nm)	Zen processed (nm)	Huygens processed (nm)
405	140	93
488	149	105

*Note*: Raw image data from the Airyscan detector were processed either using Zeiss Zen Airyscan Processing module or using Huygens software (Array Detector deconvolution module). The lowest experimental resolution was determined using the full width at half maximum of the point spread function using 100 nm diameter fluorescent beads on a coverslip, as described in Methods.

First, we validated our imaging and quantification method by co‐staining different organelles markers in HeLa cells (Figure 1B–D). We observed a robust partitioning between the *cis*‐Golgi protein, GM130, and the *trans*‐Golgi protein, GCC88 (linescan Figure 1C, top panel). The coefficient of colocalization for GM130 compared to GCC88 was 0.16, while the inverse was 0.22 (Figure 1D). The two *trans*‐Golgi proteins, GCC88 and golgin97, were spatially closer, nonetheless we could distinguish the individual markers in the TGN (linescan Figure 1C, middle panel), consistent with previous findings that these golgins are found on distinct subdomains of the TGN.^32^ The colocalization coefficients of GCC88 and golgin97 were 0.52 and 0.59. Finally, the staining of GM130 using two different primary antibodies shows a perfect colocalization (linescan Figure 1C, lower panel) with a colocalization coefficient associated was 1.0. The colocalization coefficient between Golgi proteins (GM130, golgin97 and GRASP65) and lysosomes (CD63 and LAMP1) were below 0.16. From this quantification (Figure 1D), we designed three thresholds. The *cis* versus *trans* markers defined a negligible threshold (the background). The level of colocalization of the two *trans* Golgi markers corresponded to a moderate coefficient of colocalization. Dual staining of GM130 with two different primary antibodies designed an artificial perfect colocalization, which corresponds to a perfect colocalization threshold. Our imaging and quantification methods provided the resolution to discriminate Golgi sub‐compartments and were suitable to quantify the segregation of BACE1 and APP in different Golgi sub‐compartments.

We applied our quantitative imaging methods to analyse the distribution and colocalization of APP and BACE1 in the TGN, by staining HeLa BACE1‐GFP cells for APP and the TGN Golgi marker (mix of GCC88 and golgin97; Figure 1Ea,b). For each channel, the signal was detected using automatic threshold with background substration to avoid any user bias (Figure 1Ec). A Golgi area was defined using the mask channel created by the TGN (Figure 1Ed). This TGN signal mask was then used as a filter to select the BACE1 (green) and APP (red) signals located in the TGN (Figure 1Ee). Then, the surface‐surface colocalization module of Imaris was used to calculate the volume of BACE1 and APP signals (voxels) that colocalize inside the mask TGN (Figure 1Ef). The proportion of BACE1 signal in the TGN (Figure 1Ee, green) that is coincident with the signal of APP in the TGN, based on signal volume (Figure 1Ee, red), was 0.16 and defined as “coefficient 1.” The proportion of APP signal in the TGN (Figure 1Ee, red) that is coincident with the signal of BACE1 in the TGN (Figure 1Ee, green) was 0.10 and defined as “coefficient 2.” Both colocalization coefficient values were lower than 0.16 which is the threshold defined for “negligible,” and shows spatial segregation of BACE1 and APP in the TGN.

### 2.2 Endogenous BACE1 and APP are segregated in HeLa cells

**FIGURE 2:**
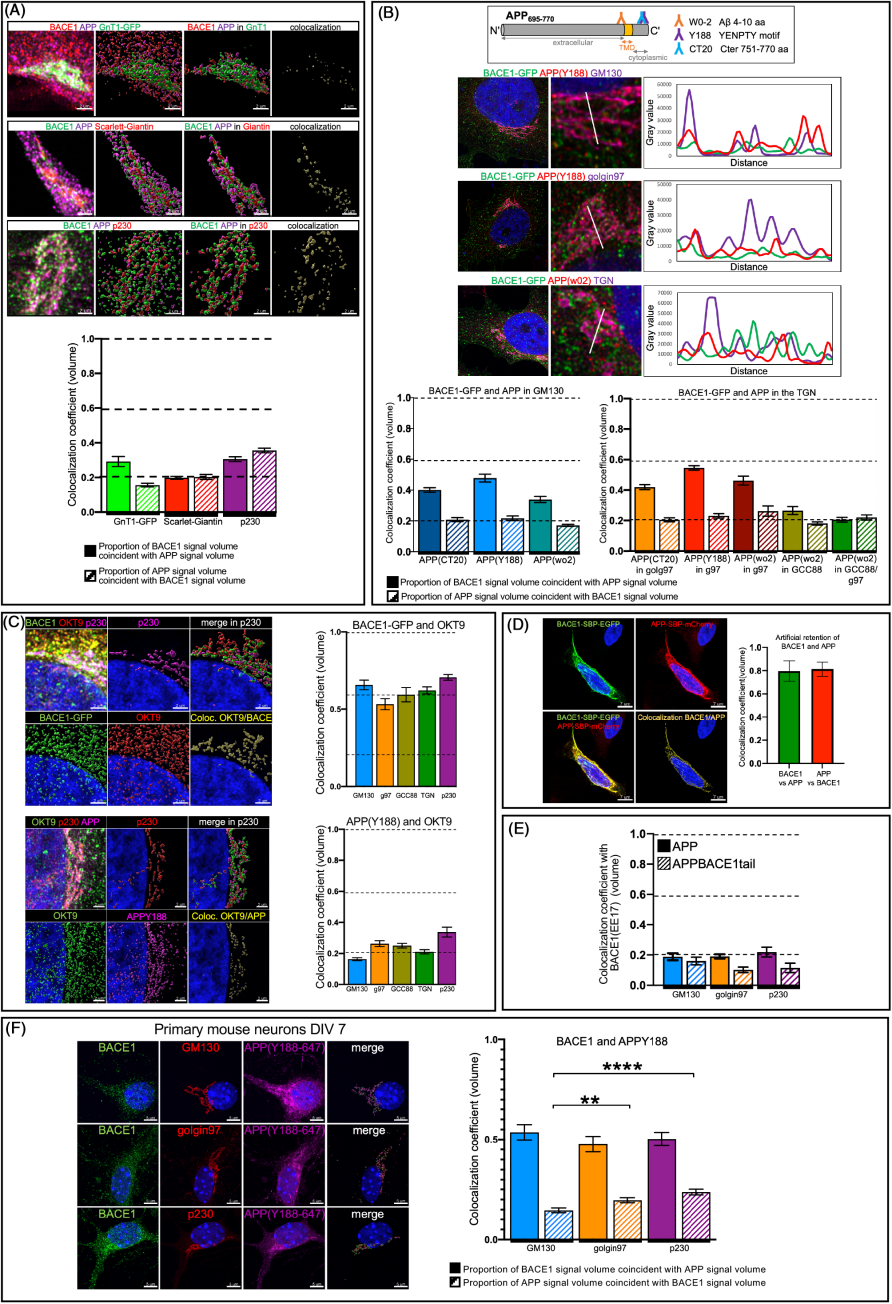
(APP) has minimal colocalization with the protease BACE1 in the Golgi. (A) Monolayers of HeLa WT cells were transfected with either GnT1‐GFP or Scarlet‐Giantin. Transfected cells and BACE1‐GFP stable cells were fixed, permeabilized and blocked. Cells were stained with anti‐BACE1 (D10E5; red or green) and anti‐APP wo2 (purple), or anti‐p230 (red) and anti‐APP wo2 (purple). Scale bar represents 4 μm. The colocalization coefficients (volume) of BACE1 and APP were calculated using Imaris. Data are represented as the mean ± SEM of three to four independent experiments (25 < *n* < 39). (B) Monolayers of HeLa cells stably expressing BACE1‐GFP was fixed, permeabilized and blocked. Cells were stained for APP using different antibodies (CT20, Y188 and wo2) and different Golgi markers anti‐GM130, anti‐golgin97, anti‐GCC88 and a mix of anti‐golgin97 (g97) and anti‐GCC88. Linescans were performed using Fiji. The colocalization coefficient (volume) of BACE1 and APP was calculated using Imaris. Data are represented as the mean ± SEM of a minimum of three independent experiments (24 < *n* < 41). (C) Monolayer of HeLa cells stably expressing BACE1‐GFP, or HeLa WT cells were fixed, permeabilized and blocked. HeLa BACE1‐GFP were co‐stained with OKT9 (red). HeLa WT cells were stained with anti‐APP(Y188) (green) and OKT9 (red). Cells were co‐stained with anti‐GM130 or anti‐golgin97 or anti‐GCC88 or anti‐GCC88 mixed with anti‐golgin97 (TGN). The colocalization coefficient (volume) of BACE1 and APP with OKT9 in each Golgi compartment were calculated using Imaris. Data are represented as the mean ± SEM of a minimum of three independent experiments (24 < *n* < 31). (D) HeLa cells were transiently transfected with Str‐Ii_BACE1‐SBP‐EGFP and Str‐Ii_APP‐SBP‐mCherry constructs for 24 h. In absence of biotin, BACE1 and APP are artificially retained in the ER thanks to their Str‐Ii hook. Monolayers of HeLa cells were fixed, then permeabilized and stained with DAPI. The colocalization coefficient (volume) of BACE‐SBP‐EGFP and APP‐SBP‐mCherry were calculated using Imaris. Data are represented as the mean ± SEM of two independent experiments (*n* = 10). (E) Monolayers of HeLa WT cells were transfected with either Str‐Ii_APP‐SBP‐EGFP or Str‐Ii_APP_BACE1tail_‐SBP‐EGFP in the presence of biotin (no retention). Cells were fixed, permeabilized and blocked. Cells were co‐stained with anti‐BACE1 (EE17) and either anti‐GM130 or anti‐golgin97 or anti‐p230. The colocalization coefficient (volume) of BACE1 and the transfected APP inside the Golgi were calculated using Imaris. Data are represented as the mean ± SEM of a minimum of three independent experiments (10 < *n* < 29). (F) Primary mouse cortical neurons were fixed at DIV7, blocked, permeabilized and co‐stained with anti‐BACE1 (D10E5; green), anti‐APP(Y88647; purple) and anti‐GM130 or anti‐golgin97 or anti‐p230 (red). The colocalization coefficient (volume) of BACE1 and APP inside the Golgi were calculated using Imaris. Data are represented as the mean ± SEM of four independent experiments (19 < *n* < 33) and analysed by unpaired, two‐tailed *t*‐tests. **p = 0.0068, ****p < 0.0001. APP, amyloid precursor protein; BACE1, β‐site amyloid precursor protein cleaving enzyme; ER, endoplasmic reticulum

The above analyses show that BACE1‐GFP is segregated from APP in the TGN when expressed heterologously. To determine, if endogenous BACE1 and APP are segregated within the Golgi stack and/or TGN, we first transfected HeLa WT cells with a Golgi stack marker, either GnT1‐GFP (also named MGAT1‐GFP) or Scarlet‐Giantin, to define the Golgi region and then stained the transfected cells with anti‐BACE1 and anti‐APP (rabbit, Y188) antibodies. Endogenous BACE1 and APP were both detected in the Golgi. However, their colocalization coefficients were around the negligible threshold (GnT1‐GFP coefficient 1 = 0.29, coefficient 2 = 0.16; Scarlet‐Giantin coefficient 1 and coefficient 2 = 0.2; Figure 2A). We stained HeLa WT cells using a human anti‐p230 antibody to mark the TGN and observed a similar segregation of the endogenous proteins BACE1 and APP (coefficient 1 = 0.31, coefficient 2 = 0.36; Figure 2A). Notably, the staining volume (voxels) of endogenous BACE1 and APP is very comparable in the different Golgi subcompartments of WT HeLa cells (Figure S2A), indicating similar levels of each cargo throughout the Golgi stack. Collectively, these data indicate that endogenous BACE1 and APP are segregated in the TGN, as for the BACE1‐GFP stable HeLa line, as well as in the Golgi stack.

Given the above findings, the BACE1‐GFP stable HeLa line represents a viable system to further investigate in detail the nature of the partitioning of these membrane cargoes in the Golgi. Next, we examined the segregation of BACE1 and APP in the BACE1‐GFP cell line with a *cis* Golgi (GM130) marker and TGN markers. We used the two TGN‐markers discussed above for Figure 1, namely golgin‐97 and GCC88, to include multi‐subdomains of the TGN. In addition, we also examined the segregation of BACE1 and APP using three different APP antibodies (CT20, Y188 and wo2), which recognize APP epitopes on the C‐terminal and the N‐terminal side of the transmembrane (TM) domain (as depicted in Figure 2B), to validate the analysis using different antibodies. The proportion of BACE1‐GFP signal in the *cis*‐Golgi compartment (GM130) that is coincident with the signal of APP (defined by Y188) was 0.48, a coefficient above the threshold but below the level of moderate colocalization. The three different APP antibodies showed a level of colocalization within the same range. On the other hand, the proportion of APP that is coincident with the BACE1‐GFP signal within the APP volume had a maximum colocalization coefficient of 0.22 (Figure 2B), a level similar to endogenous BACE1 (Figure 2A). The difference between coefficient 1 and coefficient 2 may reflect differences in abundance of BACE1 and APP in the Golgi under steady state conditions. We observed similar colocalization coefficients using the *trans*‐Golgi marker golgin97 and the APP antibodies, CT20, Y188 and wo2, and a negligible colocalization coefficient for the GCC88 Golgi sub‐compartment. To increase the TGN volume included in the analyses, we combined two TGN antibodies, golgin97 and GCC88, in the same channel, and refer to this volume of the combined markers as “TGN.” BACE1‐GFP and APP colocalization was below the threshold in the stained TGN, and suggested that the larger TGN volume gave a more accurate assessment of the extent of segregation of the total pool of BACE1‐GFP and APP. Altogether, our analyses indicated a segregation of BACE1 and APP throughout the Golgi. Interestingly, the segregation of BACE1‐GFP and APP was significantly enhanced from the *cis*‐Golgi to the TGN.

As a control for the localisation studies, we included the transferrin receptor (TfR), as both the TfR and BACE1 have been reported to be transported directly to the cell surface from the TGN,^12^, ^33^ and therefore they may be spatially co‐incident throughout the Golgi stack. BACE1‐GFP stable HeLa cells were stained with OKT9 for the TfR and the level of colocalization in the *cis* and *trans*‐Golgi compartments assessed using GM130 and golgin97/GCC88, respectively (Figure 2C). BACE1‐GFP showed a much higher level of colocalization with the TfR in the *cis*‐Golgi (0.66) than between APP and BACE1‐GFP. The colocalization between BACE1‐GFP and TfR in the TGN (combined GCC88 and golgin97 markers) is also high (0.62). The staining volume (voxels) of BACE1‐GFP and TfR is very similar in the *cis* and *trans*‐Golgi of BACE1‐GFP stable HeLa cells (Figure S2B). In contrast, APP did not colocalize with OKT9 in either the *cis*‐Golgi or the TGN with only a threshold level of co‐incidence in both compartments (0.16 for *cis*‐Golgi, 0.20 for TGN; Figure 2C). To demonstrate that our system could successfully detect perfect colocalization of APP and BACE1, we artificially blocked BACE1 and APP in the ER using the Retention Using Selective Hooks (RUSH) system.^12^, ^34^ Cargoes are retained in the ER as a consequence of the interaction between a streptavidin molecule fused to a reticulum signal (Ii) and a Streptavidin‐binding peptide (SBP) fused to the cargo. We co‐transfected BACE1‐SBP‐EGFP and APP‐SBP‐mCherry and observed a high colocalization coefficient between BACE1‐SBP‐EGFP and APP‐SBP‐mCherry (0.80 and 0.81) artificially retained in the ER (Figure 2D). This result demonstrates that our system can detect high levels of colocalization of BACE1 and APP if it occurs. Taken together, these findings support the conclusion that BACE1 and APP are segregated in the Golgi stack.

We also examined the level of colocalization of BACE1‐GFP and APP in the early endosomes (EEA1), recycling endosomes (Rab11) and lysosomes (CD63; Figure S1A). Only low levels of colocalization between BACE1‐GFP and APP were detected in the early endosomes, as revealed by line scans and a colocalization co‐efficient of 0.23, suggesting that BACE1‐GFP and APP are located to different domains of the early endosomes. The colocalization of APP and BACE1‐GFP in the recycling endosomes (Rab11) and lysosomes (CD63) was negligible.

### 2.3 The segregation of APP and BACE1 is independent of the cytoplasmic domain of the cargo

To determine if the cytoplasmic tail of APP is solely responsible for APP/BACE1 segregation, we replaced APP cytoplasmic tail in the WT APP with the BACE1 tail (referred to APP_BACE1tail_). To differentiate endogenous BACE1 from APP_BACE1tail_, we used a BACE1 antibody (BACE1 EE17) that recognized the N‐terminus of BACE1 and will recognize endogenous BACE1 but not the chimera construct (Figure 2E). We observed a negligible colocalization coefficient between BACE1 and APP_BACE1tail_ in either the *cis*‐Golgi or TGN sub‐compartments (Figure 2E). Therefore, the APP_BACE1tail_ construct is well segregated from BACE1. These results indicate that the mechanism of APP and BACE1 segregation is independent of the cytoplasmic domain of APP.

### 2.4 BACE1 and APP segregation in primary mouse neurons

In order to know if the segregation of APP and BACE1 in HeLa cells was an accurate representation of the segregation of APP and BACE1 in neurons, we applied our methods to primary mouse cortical neurons. We first checked the segregation of resident Golgi proteins (similar to Figure 1D) in primary mouse cortical neurons at 7 days in vitro (DIV7), and at DIV14, as neurons have distinctive Golgi structures at the two time periods in culture. Golgi outposts are very rare in DIV7 neurons but are present in DIV14 neurons. We were able to discriminate the *cis*‐Golgi protein GM130 and the *trans*‐Golgi proteins (p230, GCC88, golgin97) as well as two *trans*‐Golgi proteins (Figure S1C). Note that p230 seems to have a very similar localization as the other TGN golgins, GCC88 and golgin97, with a higher colocalization coefficient of the golgins compared with HeLa cells. There was perfect colocalization with double antibody staining for GM130 in the two channels with a coefficient of 1.0. Similar findings were observed in DIV14 mature primary neurons containing Golgi outposts (data not shown). To stain endogenous APP and BACE1 we used a directly conjugated antibody to APP (Y188‐AF647). Staining of HeLa cells and primary mouse neurons successfully detected endogenous BACE1 and APP when the antibody to APP (Y188‐AF647) was used (Figure S1B,D). Primary mouse neurons DIV7 were then co‐stained with anti‐BACE1, anti‐APP(Y188‐AF647) and either GM130, golgin97 or p230 (Figure 2F). We observed a moderate colocalization coefficient of BACE1 with APP, whereas there was only a low colocalization coefficient of APP with BACE1. The moderate BACE1 co‐localization coefficient with APP was persistent through the different Golgi sub‐compartments (Figure 2F). Although APP co‐localization with BACE1 was only low throughout the Golgi stack, there was significantly higher colocalization with the TGN markers, golgin97 and p230, compared to the *cis*‐Golgi marker, GM130, in DIV7 neurons (Figure 2F); however, DIV14 neurons showed similar low levels of APP colocalization with BACE1 in both *cis*‐Golgi and TGN (Figure S1E). The key finding is that only low levels of BACE1 overlap with the total pool of APP in the different Golgi regions. Notably, the staining volume (voxels) of endogenous APP in the *cis* and *trans* Golgi of primary neurons is 2–3 folder higher than endogenous BACE1 (Figure S2C), and this difference mostly likely accounts for the higher level of BACE1 overlapping with APP, and suggests the efficiency of segregation is dependent on the relative levels of the two cargoes.

### 2.5 The Golgi ribbon is not necessary for the segregation of BACE1 and APP

**FIGURE 3:**
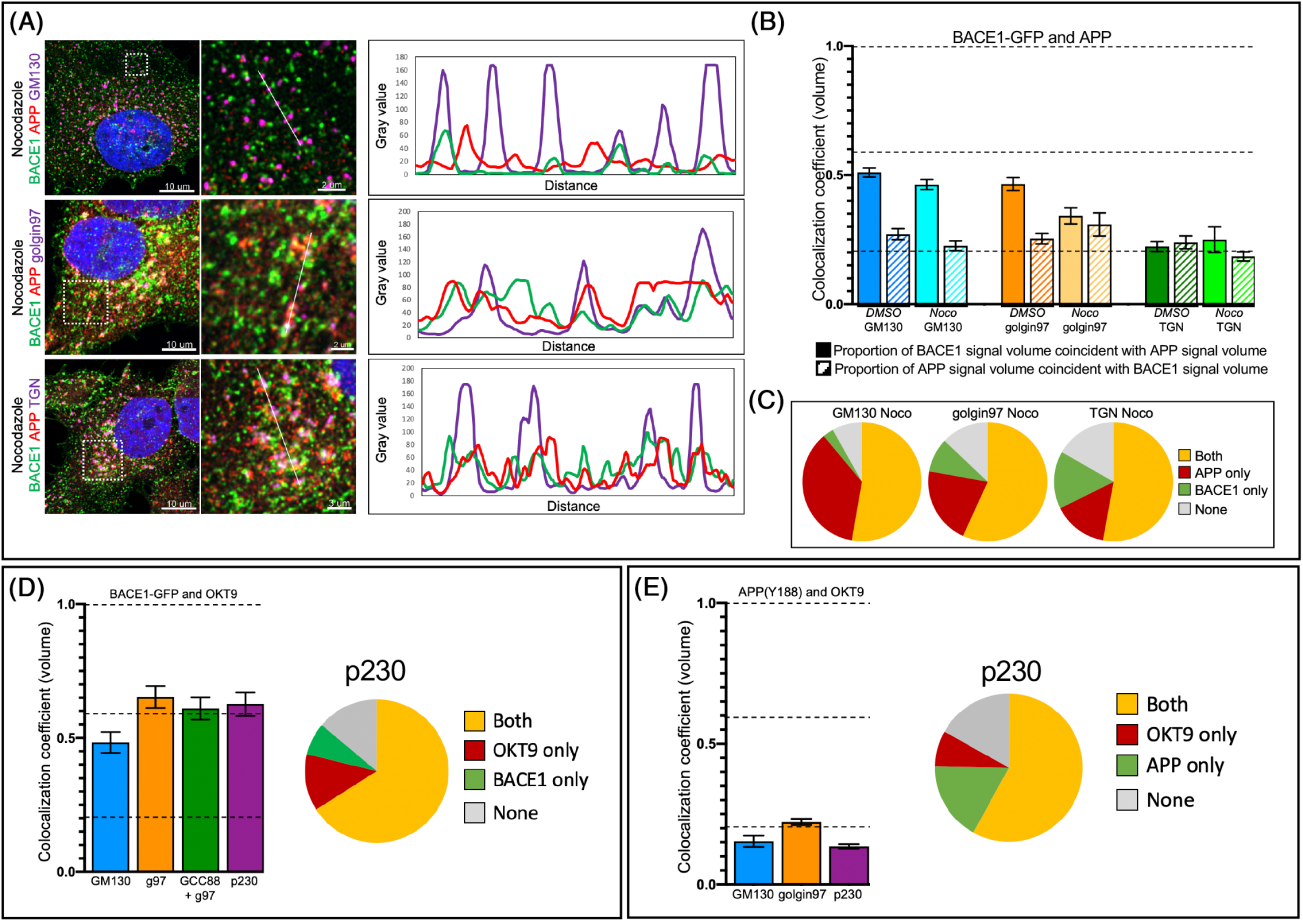
The Golgi ribbon is not mandatory for BACE1 and APP segregation. (A) Monolayers of HeLa cells stably expressing BACE1‐GFP were treated with 10 μM nocodazole for 2 h, fixed and permeabilized, and stained with anti‐APP(Y188) (red) and anti‐GM130 or anti‐golgin97 or anti‐golgin97 mixed with anti‐GCC88 (TGN; purple). Linescans were performed using Fiji. (B and C) Monolayers of HeLa cells stably expressing BACE1‐GFP were treated with 0.1% DMSO (carrier) or 10 μM nocodazole for 2 h, fixed and permeabilized and stained with anti‐APP and anti‐GM130 or anti‐golgin97 or anti‐golgin97 mixed with anti‐GCC88 (TGN). (B) The colocalization coefficient (volume) of BACE1‐GFP and APP were calculated using Imaris. Data are represented as the mean ± SEM of three independent experiments (16 < *n* < 29). (C) Co‐occurrence of BACE1‐GFP and APP in Golgi ministacks. Data are represented as the mean of three independent experiments (16 < *n* < 29). Pie charts represent the percentage of the co‐occurrence of BACE1‐GFP and APP in individual ministacks for each different condition. Golgi ministacks can contain only APP (red), only BACE1 (green), APP and BACE1 (orange) or can be empty for BACE1 and APP (gray). (D) Monolayers of HeLa cells stably expressing BACE1‐GFP were treated with 10 μM nocodazole for 2 h at 37°C, fixed and permeabilized, and stained with OKT9 and anti‐GM130 or anti‐golgin97 or anti‐golgin97 mixed with anti‐GCC88 (TGN) or anti‐p230. The colocalization coefficient (volume) of BACE1‐GFP and OKT9 were calculated using Imaris. Data are represented as the mean ± SEM of three independent experiments (21 < *n* < 25). Pie charts represent the percentage of the co‐occurrence of BACE1‐GFP and OKT9 in individual Golgi ministacks for each different condition. Ministacks can contain only OKT9 (red), only BACE1 (green), BACE1 and OKT9 (orange) or can be empty for BACE1 and OKT9 (gray). (E) Monolayers of HeLa WT cells were treated with 10 μM of nocodazole for 2 h at 37°C, fixed and permeabilized, and stained with anti‐APP(Y188), OKT9 and anti‐GM130 or anti‐golgin97 or anti‐p230. The colocalization coefficient (volume) of APP(Y188) and OKT9 were calculated using Imaris. Data are represented as the mean ± SEM of three independent experiments (14 < *n* < 25). Pie charts represent the percentage of the co‐occurrence of APP(Y188) and OKT9 in individual Golgi ministacks for each different condition. Ministacks can contain only OKT9 (red), only APP(Y188) (green), APP(Y188) and OKT9 (orange) or can be empty for APP(Y188) and OKT9 (gray). APP, amyloid precursor protein; BACE1, β‐site amyloid precursor protein cleaving enzyme

The Golgi stacks in HeLa cells and neurons exist as a complex ribbon structure where the individual stacks are fused via their lateral faces. In order to examine whether the organization of the Golgi as ribbon structure contributed to the segregation of BACE1 and APP, we took advantage of the microtubule depolymerising agent nocodazole, which dissociates the Golgi ribbon and disperses individual Golgi stacks throughout the peripheral cytoplasm.^35^, ^36^ HeLa‐BACE1‐GFP stable cells were treated with nocodazole and, to define the dispersed Golgi puncta, we stained nocodozale‐treated cells with markers of the *cis*‐Golgi (GM130) and the TGN (golgin97, GCC88 and p230). The majority of Golgi puncta stained with both *cis*‐Golgi and TGN markers (Figure S3A), indicating the puncta were intact Golgi mini‐stacks. The level of colocalization of BACE1 and APP in the Golgi stacks was assessed. The loss of the Golgi ribbon and conversion into dispersed Golgi ministacks (Figure 3A) did not result in the loss of segregation of BACE1‐GFP and APP in the Golgi (Figure 3A,B). Since it was possible that BACE1‐GFP and APP might be segregated in different ministacks, we quantified the co‐occurrence of BACE1‐GFP and APP in the different Golgi ministacks. We found that half of the ministacks were composed of both BACE1‐GFP and APP (Figure 3C, yellow shading). In the remaining ministacks, around 15% have only BACE1‐GFP, 15% only APP and 15% have neither BACE1‐GFP or APP. We inhibited gamma‐secretase activity with DAPT to determine if the processing of APP is responsible for the finding that some ministacks have only one of the two membrane proteins. Using anti‐APP(Y188) to detect APP, which recognizes the YENPTY sorting motif in the cytoplasmic tail of APP, there was no difference in the relative distribution of BACE1 and APP in the Golgi ministacks; hence, APP processing did not account for the finding that some ministacks have only one of the two membrane proteins (Figure S3B–D). Inhibition of BACE1 activity with the β‐secretase/BACE1 inhibitor C3 also did not dramatically change the relative distribution of BACE1 and APP in the Golgi ministacks (Figure S3E). Therefore, the negligible colocalization of BACE1‐GFP and APP is not a result of segregation of BACE1 and APP in different single ministacks, rather spatial segregation of BACE1 and APP within the membranes of the same Golgi ministack unit. Moreover, BACE1‐GFP and TfR (OKT9) colocalization was conserved in the absence of a Golgi ribbon (Figure 3D) with a high co‐occurrence in the ministacks. Both APP and TfR (OKT9) were also found in the majority of the individual ministacks, however, there was no colocalization of these two membrane proteins (Figure 3E).

These results demonstrate the Golgi ribbon was not essential for the segregation of BACE1 and APP; rather BACE1 and APP segregation was observed from the *cis*‐Golgi indicating that segregation was occurring either prior to Golgi entry or immediately on delivery to the Golgi.

### 2.6 Early segregration of BACE1 and APP in the ER

**FIGURE 4:**
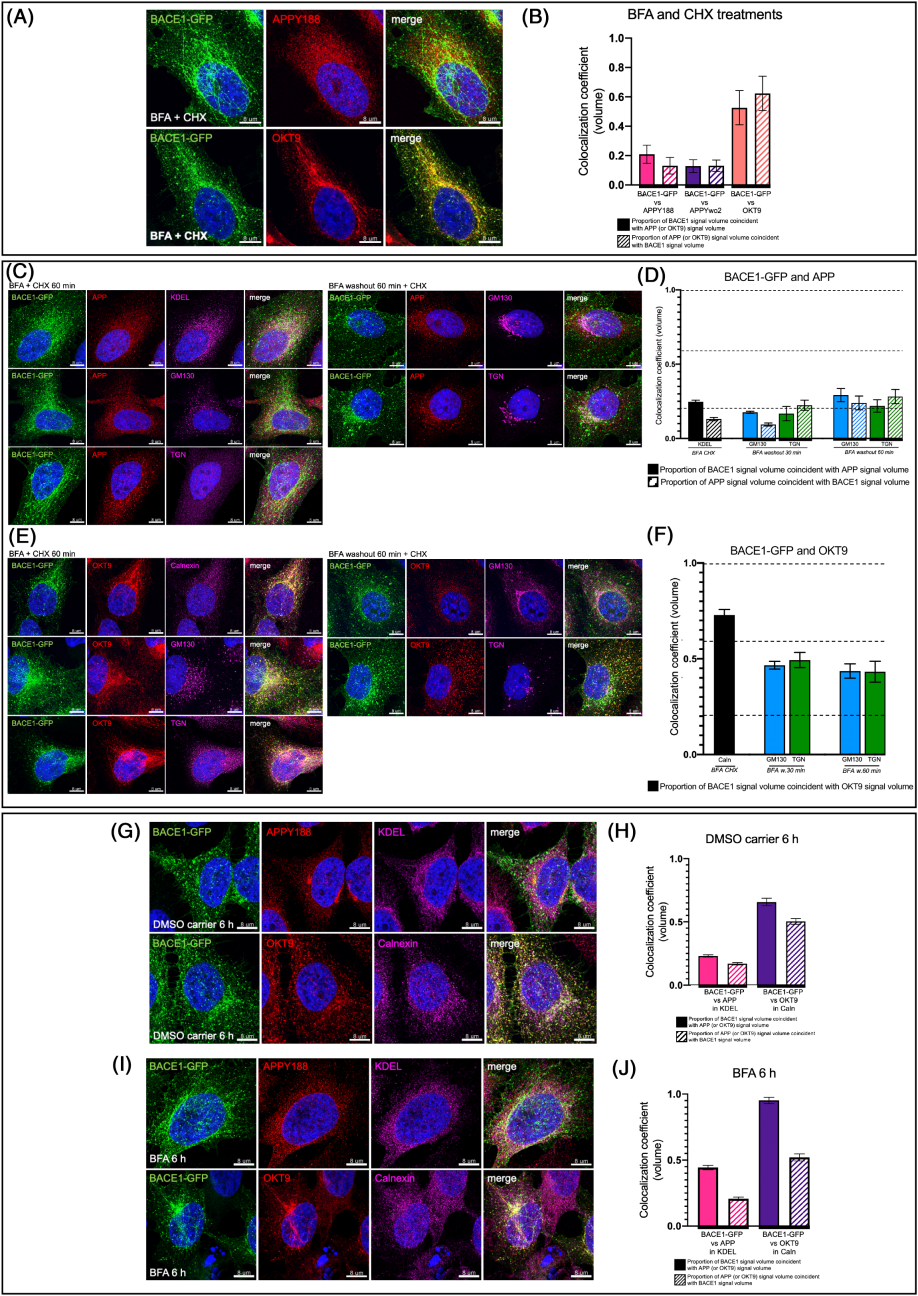
Segregation of BACE1 and APP in the ER after BFA treatment. (A and B) Monolayers of HeLa cells stably expressing BACE1‐GFP were treated with 1 μM BFA and 100 μg/mL of CHX for 1 h at 37°C, monolayers were fixed and permeabilized, and stained with anti‐APPY188 or OKT9 (anti‐TfR) and the nuclei stained with DAPI (blue). The colocalization coefficient (volume) of BACE1‐GFP and APP was calculated using Imaris without mask. Data are represented as the mean ± SEM of four independent experiments (23 < *n* < 50). (C and D) Monolayers of HeLa cells stably expressing BACE1‐GFP were treated with 1 μM BFA and 100 μg/mL of CHX for 1 h at 37°C. Monolayers were washed extensively in PBS (×10 times) to wash‐out the BFA, and chased for 30 and 60 min in the presence of CHX. At each chase time, monolayers were fixed and permeabilized, and stained with anti‐APP and anti‐KDEL, or anti‐GM130 or anti‐golgin97 mixed with anti‐GCC88 (TGN). The colocalization coefficient (volume) of BACE1‐GFP and APP was calculated within the KDEL, GM130 or TGN mask using Imaris. Data are represented as the mean ± SEM of four independent experiments (21 < *n* < 28). (E and F) Monolayers of HeLa cells stably expressing BACE1‐GFP were treated as for C and D, and cells stained with OKT9 and anti‐calnexin, or anti‐GM130 or anti‐golgin97 mixed with anti‐GCC88 (TGN). The colocalization coefficient (volume) of BACE1‐GFP and OKT9 was calculated within the calnexin (Caln), GM130 or TGN mask using Imaris.. Data are represented as the mean ± SEM of three independent experiments (13 < *n* < 17). (G–J) Monolayers of HeLa cells stably expressing BACE1‐GFP were treated with 1 μM BFA (or DMSO carrier) for 6 h at 37°C, monolayers were fixed and permeabilized, and stained with anti‐APPY188 or OKT9 (anti‐TfR) and anti‐KDEL or anti‐calnexin, and the nuclei stained with DAPI (blue). The colocalization coefficient (volume) of BACE1‐GFP and APP or BACE1‐GFP and OKT9 were calculated within the KDEL or calnexin (Caln) mask using Imaris. Data are represented as the mean ± SEM of three independent experiments (28 < *n* < 43). APP, amyloid precursor protein; BACE1, β‐site amyloid precursor protein cleaving enzyme; BFA, brefeldin A; ER, endoplasmic reticulum

Endogenous BACE1 and APP are synthesized in the ER before transport to the Golgi. To determine whether is there spatial segregation within the ER environment, we treated BACE1‐GFP stable HeLa cells with brefeldin A (BFA), which blocks the activation of Arf1 and results in reabsorption of Golgi membrane proteins back to the ER,^37^, ^38^ together with cycloheximide (CHX) to inhibit protein translation (Figure 4A). Following BFA treatment, BACE1‐GFP and APP staining was typical of a reticular ER staining pattern. Strikingly, BACE1 and APP were segregated following BFA treatment with only minimal colocalization (Figure 4A,B). We also used KDEL and calnexin as markers of the ER. Within the KDEL mask, BACE1 and APP were clearly segregated in the ER (Figure 4C,D). In contrast, BACE1 overlapped extensively with TfR in the ER of BFA treated cells (Figure 4A); and showed a high level of colocalization within the ER mask, calnexin (Figure 4E,F). These results clearly demonstrate the differential subdomain location of BACE1 and TfR from APP as early as the ER in the anterograde pathway.

BFA was then washed out and the reformation of a compact Golgi was monitored. One hour after BFA washout, a GM130‐positive compact Golgi was detected (see Golgi staining in Figure 4C,E), and, in addition, there was a substantial reduction of the ER reticular staining pattern for BACE1 and APP and a relocation of both cargos to the Golgi region. The colocalization coefficient between BACE1 and APP in the reformed Golgi was similar to untreated cells (Figure 4C,D). In comparison, BACE1 colocalization with TfR (OKT9) was even higher in the ER in the presence of BFA than the Golgi (see Figure 2C). The level of colocalization of BACE1‐GFP and TfR 1 h after BFA washout was similar to untreated cells with a coefficient of 0.44 in GM130 and 0.43 in the TGN (Figure 4F).

To determine if newly synthesized BACE1 and APP may segregate in the ER we treated cells for an extended period in BFA (6 h) without CHX to allow the synthesis of new protein during this period. There was a similar level of segregation of APP and BACE1 (Figure 4G–J), compared with BFA treatment in the presence of CHX, suggesting that nascent cargo and relocalized cargoes are able to segregate in the ER. Collectively these findings indicate that BACE1 and APP are segregated very early in the anterograde pathway.

**FIGURE 5:**
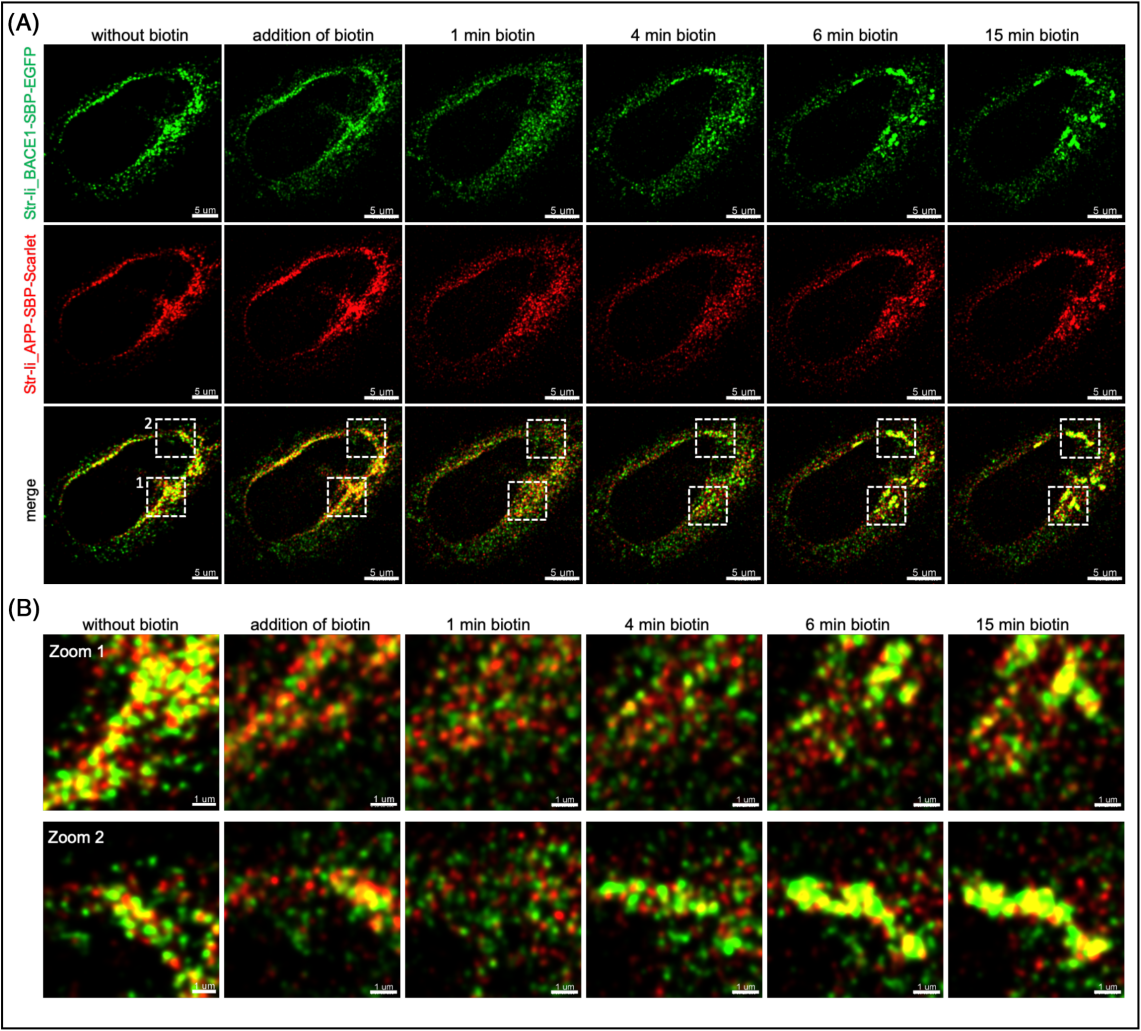
Early segregation of newly synthetized APP and BACE1 in the endoplasmic reticulum. (A and B) HeLa cells were transiently co‐transfected with Str‐Ii_BACE1‐SBP‐EGFP and Str‐Ii_APP‐SBP‐Scarlet constructs for 12 h. In absence of biotin, BACE1 and APP are artificially retained in the ER by the Str‐Ii hook (without biotin). Biotin was added just before the start of the movie (= addition of biotin) and images at the indicated time points after biotin addition are shown. Images were taken every 10 s for a duration of 90 cycles (15 min). The boxed regions in the merge in A are shown enlarged in B. Scale bars represent 5 μm or 1 μm, as indicated. APP, amyloid precursor protein; BACE1, β‐site amyloid precursor protein cleaving enzyme; ER, endoplasmic reticulum

To directly determine whether nascent APP and BACE1 exit the ER together or are segregated, we performed SR dual color fast live cell imaging using the RUSH system to simultaneously track the early trafficking itinerary of both APP and BACE1 from the ER to the Golgi (Figure 5). In the absence of biotin, APP‐SBP‐Scarlet and BACE1‐SBP‐EGFP are both articifically retained in the same ER sub‐compartment and a considerable overlap of APP and BACE1 is observed (Figure 5A), consistent with results above. One minute after, the release of APP and BACE1 (1 min biotin) following biotin addition, distinct punctate and/or vesicular structures were observed and the majority of these structures were bearing either APP‐SBP‐Scarlet or BACE1‐SBP‐GFP, and very few of these discrete puncta were detected with both cargoes (Figure 5A,B). We know from a previous study^12^ that BACE1 reaches the Golgi 10 to 15 min after the addition of biotin. We observed a segregation of APP and BACE1 from 1 min of biotin to 6 min (Figure 5B). These Scarlet and GFP labeled puncta are likely to represent either ER exit sites or ER derived transport carrriers. These data are consistent with the BFA experiments, showing a segregation of APP and BACE1 in the ER after BFA treatment. Further work is needed to elucidate the mechanism of the early segregation of APP and BACE1 in the anterograde trafficking. Taken together, these findings suggest that APP and BACE1 are segregated and transported independently from the ER.

## 3 DISCUSSION

**FIGURE 6:**
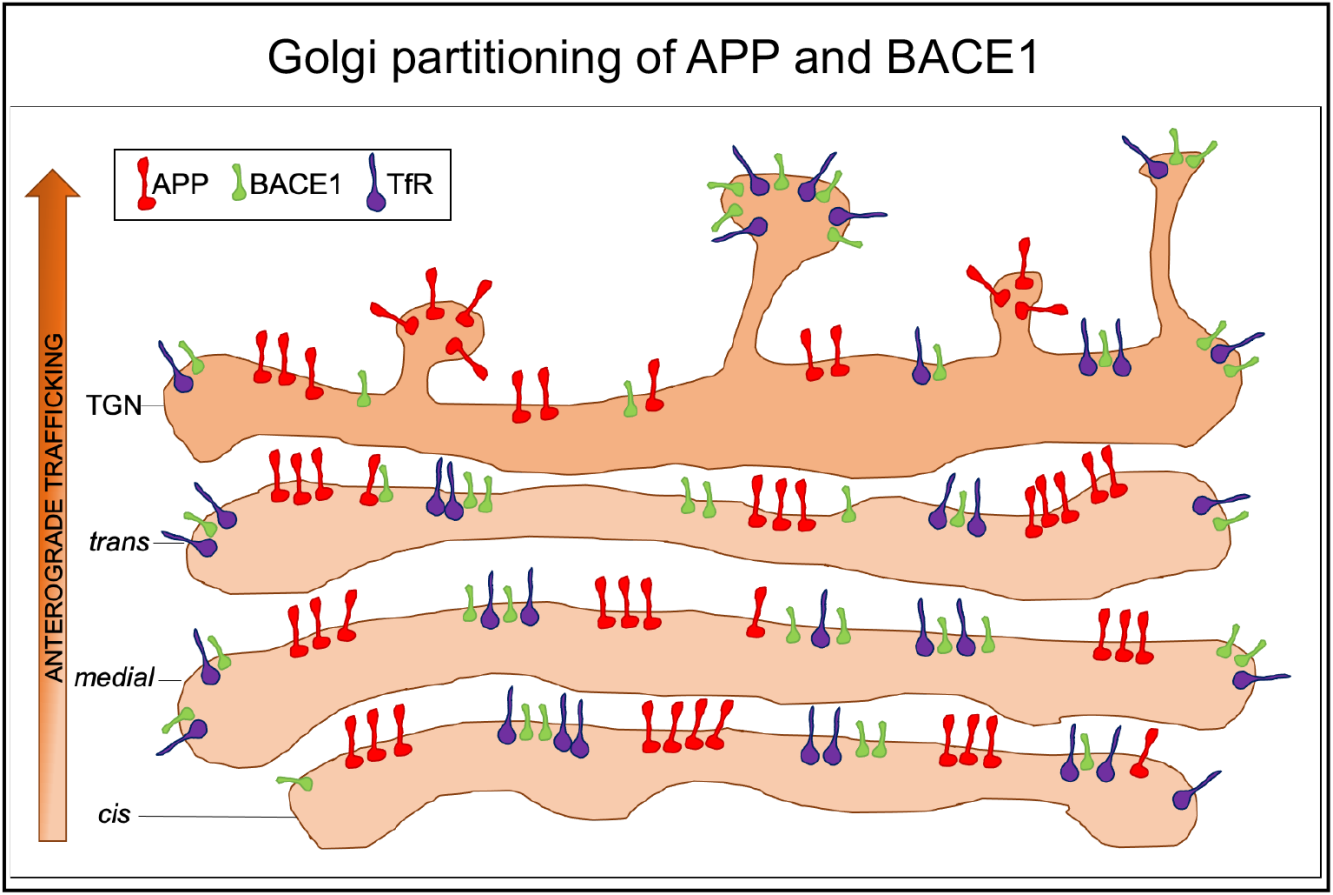
Model depicting the segregation of membrane cargoes in the Golgi stack. Model showing the partitioning of APP, BACE1 and TfR in a Golgi stack. The model proposes that APP is partitioned from BACE1 and TfR into subdomains of the *cis*‐Golgi within individual stacks and the majority of APP remains segregated from BACE1 throughout the Golgi stack and TGN. At the TGN, APP and BACE1 exit the Golgi in different transport carriers. Only low levels of BACE1 is in physical proximity to APP. APP, amyloid precursor protein; BACE1, β‐site amyloid precursor protein cleaving enzyme

The membrane cargoes APP and the β‐secretase BACE1 follow the same early anterograde route through the ER and the Golgi apparatus. The traditional view is that cargo proteins are mixed together as they pass through the Golgi stack and are sorted and segregated in the TGN for distinct populations of post‐Golgi transport pathways for delivery to the PM, lysosomes and endosomes. As APP processing and Aβ production can occur in the secretory pathway, a key question is how the level of APP processing is regulated and whether APP and BACE1 are segregated prior to their exit from the Golgi. APP and BACE1 are known to be shunted into different transport pathways from the TGN in various cell types including neurons, which indicates sorting pathways of these membrane cargoes in the secretory pathway are conserved across cell types. However, it was unknown whether segregation of APP and BACE occurs prior to signal‐adaptor protein mediated sorting in the TGN. By coupling SR microscopy with quantification analysis, here we demonstrate that (1) endogenous APP and BACE1 are segregated in HeLa cells and primary mouse neurons in the early Golgi apparatus; (2) in contrast, BACE1 and TfR show substantial overlap in the Golgi stack and TGN; (3) segregation of APP and BACE1 was observed within individual Golgi ministacks indicating partitioning of the two cargoes within the same membrane compartment as depicted in the model presented in Figure 6; (4) segregation of APP and BACE1 is maintained after BFA treatment and transport back to the ER compartment, (5) newly synthesized APP and BACE1 exit the ER in distinct transport carriers and (5) segregation of APP from BACE1 does not depend on a specific cytoplasmic sorting signal. Importantly, we demonstrated that heterologously tagged and endogenous BACE1 and APP are segregated in the Golgi, which supports the relevance of our findings under physiological conditions.

To define the Golgi compartments, we established a quantitative analysis using SR Airyscan detector coupled with deconvolution analysis to give a lateral resolution of ~90 nm, a resolution sufficient to distinguish individual cisternae and potential subdomains within individual cisternae. Using a range of markers, there was a clear definition of either *cis*‐ or medial‐Golgi markers and *trans* Golgi markers and also differences between different individual TGN markers which define distinct subdomains of the TGN. We used the Golgi markers to define the specific region of the Golgi and then assessed the level of overlap of individual cargoes within that Golgi region. A number of controls were included to validate the analysis. We showed that “perfect” co‐localization was detected with two different antibodies to a single Golgi marker and also for APP and BACE1 if both were restricted to the same membrane microenvironment by binding to same membrane protein, namely the hook of the RUSH system, which formally showed our analysis could reflect a high level of co‐localization when it occurs. In addition, the inclusion of TfR demonstrated that close proximity of different cargoes could be detected in the Golgi stack and that BACE1 was not the only membrane cargo segregated from APP. The dual staining analysis included consideration for the percentage of each component within the total pool to account for differences in the abundance of each component within the compartment, for example we calculated both the percentage of total APP that overlapped with BACE1 and the percentage of BACE1 which overlapped with APP. For the most part there were similar levels of overlap, but with some exceptions, notably the endogenous proteins in primary neurons. As APP is highly expressed in neurons,^39^ and we showed that the abundance of APP in the Golgi is higher than BACE1, it is likely that the difference refers to different levels of expression of each cargo. Another consideration of the relative abundance of APP and BACE1 within the Golgi under steady state distribution is residency time of each cargo within this compartment, which is currently not well defined.

In the TGN, APP and BACE1 are shunted into pathways regulated by AP‐4 and AP‐1, respectively. Silencing of AP‐1 or AP‐4 is associated with increased APP processing in the TGN,^9^, ^12^, ^25^ however, the increase in BACE1 cleavage was relatively modest, findings which indicated there could be additional mechanisms to partition APP and BACE1 besides AP‐sorting signal interactions. The possibility that cargoes moving along the secretory pathway can be segregated has been suggested over the past 15–20 years from a variety of different systems. Large cargoes and protein aggregates have been proposed to be restricted to the rims of the Golgi cisternae, based on EM and SR optical microscopy, whereas conventional membrane cargoes are detected in the interior of the cisternae.^40^, ^41^ Furthermore, membrane cargoes are sorted from the resident membrane glycosyltransferases during transit through the stack, which implies mechanisms for partitioning different membrane proteins of similar size. Segregation of newly synthesized proteins within the Golgi has been shown for two endolysosomal proteins^33^ and for cargoes destined for the apical and basolateral surfaces of epithelial cells.^30^ An early report of segregation of two cargoes in the ER of chondrocytes,^42^ implies there may be multiple locations in the ER and Golgi for segregation of cargoes.

One possibility to account for cargo segregation within the Golgi stack is for different cargoes to be transported through separate Golgi stacks and then converge at the TGN. Indeed, in *Drosophila* where Golgi stacks are independently dispersed throughout the cytoplasm, and three different cargoes have been shown to be transported via distinct subpopulations of Golgi stacks.^43^ By dispersing the Golgi ribbon into Golgi stacks with nocodazole, we demonstrated this was not the case for APP and BACE1 and from quantitation analyses, we demonstrated that the majority of APP and BACE share the same individual Golgi stacks. Hence, the separation of the two membrane cargoes was within the same membrane compartment.

Analysis of APP/BACE1 chimeric molecules demonstrated that the cytoplasmic domain was not responsible for this segregation and suggests that multiple domains may be involved. Of relevance is that multiple domains are responsible for the correct location of resident Golgi proteins within the stack.^44^, ^45^ The mechanisms responsible for the segregation of BACE1 and APP are unknown at this stage. One possibility is that lipid subdomains contribute to the segregation process; cholesterol/glycosphingolipid domains of Golgi membranes are known to play an important role in the organization of the Golgi and have been implicated in the partitioning of glycoprotein cargoes which are destined for the apical and basolateral surfaces in epithelial cells.^46^, ^47^ Multiple mechanisms may be involved to partition BACE1 and APP which incorporate lipid mediated sorting of membrane proteins together with specific interactions with the luminal and/or cytoplasmic domains.

Our findings also indicate that APP and BACE1 can be partitioned within an ER environment. Following BFA treatment and retrograde transport of Golgi components back the ER, APP/BACE1 do not colocalize whereas BACE1 and TfR are colocalized. However, as the Golgi lipid bilayers are also transported back to the ER, it is possible that the alterations in the ER lipid composition of the mixed ER/Golgi compartment may contribute to this segregation. Therefore, the BFA experiments do not distinguish whether newly synthesized APP and BACE1 are segregated prior to leaving the ER or on arrival at the *cis*‐Golgi. Using the RUSH system and dual expression of APP‐SBP‐Scarlet and BACE1‐SBP‐GFP, we observed segregation of the two cargoes very early upon biotin addition. The distinct structures APP‐SBP‐Scarlet and BACE1‐SBP‐GFP is consistent with independent exit sites and/or transport in distinct transport carriers. These findings indicate that the partitioning of the two cargoes occurs prior to arrival at the *cis*‐Golgi. To date, there have been very limited studies analysing transport of two membrane cargoes simultaneously from the ER, and the findings from this study suggest that cargo sorting from the ER may be under appreciated. In this respect it is of interest that GPI anchored proteins have been proposed to be segregated into a distinct population of vesicles upon exit from the ER^48^ which may be driven by the selection of specific cargoes by COPII vesicles at the ERES before they move toward the Golgi.^49^ Given these previous reports and our findings here, the quantitation analysis using SR microscopy developed in this study now has wide application to analyse the segregation of a range of cargoes in the secretory pathway.

Our findings that BACE1 and APP are segregated from the ER and in the early Golgi is important for understanding the regulation APP processing and also highlights a principle which could apply to other cargoes. Knowledge of the mechanisms of anterograde transport of APP and BACE1 is also relevant for the understanding the molecular basis of dysfunctional trafficking associated with neurodegenerative diseases. Alterations in the morphology of the Golgi is hallmark of AD, and alterations in lipid metabolism, especially cholesterol, is associated with elevated Aβ production. An important consideration for future studies is the impact of the alterations in Golgi architecture and lipid metabolism in AD on BACE1 and APP trafficking, APP processing and Aß production and secretion.

## 4 METHODS

### 4.1 Cell culture, RNAi, transient transfection and treatments

HeLa wild‐type cells and HeLa BACE1‐GFP stable cell line were grown at 37°C with 10% CO_2_ in Dulbecco's Modified Eagle's medium (DMEM, high glucose, L‐glutamine, Gibco LifeTechnologies) supplemented with 10% vol/vol fetal calf serum (FCS; Life Technologies, Thermo Fisher Scientific), 2 mM L‐glutamine, 100 U/μl penicillin and 0.1% wt/vol streptomycin (Life Technologies; complete DMEM/C‐DMEM). HeLa cells were verified by genomic sequencing and cells routinely tested for mycoplasma contamination using MycoAlert™ Mycoplasma Detection Kit (Lonza, Switzerland). HeLa BACE1‐GFP cell line was generated as previously described^12^ and maintained in C‐DMEM supplemented with 1 mg/mL Geneticin (G418 sulfate, 50 mg/mL, Life Technologies, Thermo Fisher Scientific, #10131035). Cells were transfected with calcium phosphate 24 to 48 h before observation.^50^ BACE1‐mCherry, Str‐Ii_APP_BACE1tail_‐SBP‐EGFP and GnT1‐GFP constructs were generated in our laboratory. The Scarlet‐Giantin construct (Addgene #85048) was gifted from Dorus Gadella (University, country). RUSH plasmids coding for Str‐Ii_BACE1‐SBP‐EGFP and Str‐Ii_APP‐SBP‐mCherry were from Gaelle Boncompain and Franck Perez (Institut Curie, Paris, France).^12^ Str‐Ii_APP‐SBP‐GFP was constructed by swapping the mCherry tag for a GFP tag using the restriction SBF1 and Fse1. Str‐Ii_APP‐SBP‐Scarlet was constructed by swapping the mCherry tag for a Scarlet tag using the restriction SBF1 and Fse1.

Depolymerization of microtubules was performed in DMEM containing 10 μM nocodazole (Sigma‐Aldrich) or (%) DMSO as a carrier control for 1 h at 37°C. γ‐secretase activity was inhibited with 250 nM N‐(N‐[3,5‐difluorophenacetyl]‐L‐alanyl)‐S‐phenyglycine t‐butyl ester (DAPT; D5942, Sigma‐Aldrich, Merck) for 16 h at 37°C. BACE1 activity was inhibited with 2 μM ß‐secretase/BACE1 inhibitor C3 (Calbiochem, Merck) for 16 h at 37°C. Release of the RUSH cargoes in the intracellular trafficking is induced by addition of 40 μM final concentration biotin as described previously.^34^

### 4.2 Primary mouse cortical neuronal cultures

All experiments carried out on animals (Ethics ID 1613960/1914968.1) were in accordance with animal ethics guidelines and approved by the Animal Ethics Committee, the University of Melbourne. Primary cortical neurons were prepared from the collected embryos of pregnant mice (C57BL/6) at gestational day 15–16 and cultured as previously.^51^ Primary cortical neurons were plated at a density of 0.8 × 10^5^ cells/well (24‐well plate) in poly‐D‐lysine (P0899, Sigma Aldrich) coated coverslips, and cultured in neurobasal medium supplemented with 2.5% B‐21 (MACS® NeuroBrew® ‐21, Miltenyi Biotec, 0.25% GlutaMAX, and 100 U/μl penicillin and 0.1% streptomycin (complete NBM; Life Technologies).

### 4.3 Antibodies

Primary antibodies used in this study are rabbit polyclonal anti‐GCC88 (Luke et al. 2003), rabbit monoclonal anti‐GM130 (EP892Y, ab52649), mouse monoclonal anti‐GM130 (Clone 35, #610823 BD Science), mouse monoclonal anti‐EEA1 (Clone 14, #610456 BD Science), rat monoclonal anti‐LAMP1 (Clone 1D4B, #553792, BD Sciences), mouse monoclonal anti‐Rab11 (Clone 47, #610656, BD Sciences), mouse monoclonal anti‐CD63 (MX‐49.129.5, #sc‐5275, Santa Cruz Biotechnology), mouse monoclonal, anti‐golgin97 (CDF4, #A21270, Thermo Fisher Scientific), rabbit polyconal anti‐golgin97 (#ab84340, Abcam), rabbit polyclonal anti‐GRASP65 (#ab30315, Abcam), human autoantibody to p230/golgin‐245,^52^ mouse monoclonal anti‐APP wo2 (#MABN10, Merck Millipore), rabbit monoclonal anti‐APP Y188 (#ab32136, Abcam), rabbit polyclonal anti‐APP (C20, Merck #171610), rabbit monoclonal anti‐APP188‐647 (#ab199549, Abcam), rabbit monoclonal antibody to β‐secretase (BACE1; D10E5, #5606, Cell Signalling Technology) and rabbit monoclonal anti‐BACE1 (Clone EE17, #B0681 Sigma), monoclonal antibodies to human transferrin receptor (TfR/OKT9)^53^ were purified from supernatants from a hybridoma.

Conjugated secondary antibodies used for immunofluorescence in this study are goat anti‐mouse IgG Alexa Fluor 488 nm, goat anti‐mouse IgG Alexa Fluor 568 nm, goat anti‐mouse IgG Alexa Fluor 647 nm, goat anti‐rabbit IgG Alexa Fluor 488 nm, goat anti‐rabbit IgG Alexa Fluor 568 nm, goat anti‐rabbit IgG Alexa Fluor 657 nm, goat anti‐human IgG Alexa Fluor 568 nm, goat anti‐human IgG Alexa Fluor 657 nm (Invitrogen, Thermo Fisher Scientific).

### 4.4 Immunofluorescence

Monolayers of cultured mammalian cells and primary mouse cortical neurons were fixed in 4% paraformaldehyde (PFA; Wako Pure Chemical Industries) for 10 min at room temperature (RT), permeabilized with 0.1% vol/vol Triton X‐100/PBS for 4 min at RT, and blocked in blocking solution (5% vol/vol FCS and 0.02% vol/vol sodium azide, in PBS) for 30 min to reduce non‐specific binding. All staining was conducted using the above method except for Rab11. For Rab11 staining, cells were fixed with 10% trichloracetic acid on ice for 15 min, quenched in 30 mM glycine/PBS for 10 min at RT, and then permeabilized and blocked as above. Cultured cells were stained with antibodies diluted in blocking solution, except for mouse anti‐Rab11 diluted in Can Get Signal immunoreaction enhancer solution A (Toyobo Life Science) for 4 h at RT or overnight at 4°C. Coverslips were washed in PBS and stained with fluorophore‐conjugated secondary antibodies diluted in blocking solution for 1 h at RT. For staining with APPY188‐647, monolayers were washed extensively, fixed in PFA for 3 min at RT and stained with APPY188‐647 for 4 h at RT or overnight at 4°C. Coverslips were washed in PBS and mounted in Mowiol (10% wt/vol Hopval 5–88, 25% wt/vol glycerol, 0.1 M Tris in milli‐Q water) containing Dapi (#D9542, Sigma‐Aldrich) on a microscope glass slide.

### 4.5 Microscopy

Images were acquired with a laser confocal scanning microscope Zeiss LSM880 with Airyscan (Carl Ziess) equipped with a 63 × 1.4 NA Plan‐Apochromat (420782‐9900‐799) oil immersion objective and 32 hexagonal array Airyscan detectors. Images were collected sequentially for multi‐color imaging using SR Airyscan mode, which sets the pixel size to 35 nm, an optimal sampling rate (Nyquist‐Shannon sampling) for deconvolution. Images were acquired as z‐stacks of 8–11 sections at 0.159 μm *per* section z‐step, the Nyquist‐Shannon sampling rate for axial dimension. Image acquisitions were performed using Zeiss Zen software (v2.3 SP1).

Time‐lapse acquisitions were performed using the same system at 37°C in a thermostat‐controlled chamber. Cells were incubated in Leibovit's medium (Life Technologies). Images were collected sequentially for multi‐color imaging using SR Airyscan mode with one stack every 10 s for 90 cycles. One picture was acquired before the addition of biotin (=without biotin image). Then, biotin was added in the well and the acquisition started few seconds after (=addition of biotin image).

### 4.6 Deconvolution, quantification of microscopy images and generation of graphs and statistics

The raw image data taken using Airyscan detector (containing 32‐phase images) were processed either using the instrument acquisition software (Zeiss Zen Airyscan Processing module) or using Huygens software (Array Detector deconvolution module). We compared both methods by measuring the lowest experimental resolution we could achieve using each software (Table 1). The resolution was evaluated using the full width at half maximum (FWHM) of point spread function (PSF) using 100 nm diameter fluorescent beads (FluoSpheres; Invitrogen) on a coverslip. Beads images were taken with the same acquisition setting as described above. The FWHM was measured using Huygens PSF Distiller. The lateral resolution measured was better using Huygens Processing. This led us to use Huygens Processing for the current study. Raw Airyscan images were deconvolved using Huygens Professional Deconvolustion Software (v21.04, Scientific Volume Imaging) using a conservative mode and theoretical PSF. Colocalization analysis was performed using Imaris software (v9.5.1, Oxford Instruments) by applying Surface‐surface colocalization XT. Graphs and statistical analyses were realized using Prism 9 (v9, GraphPad). Data are represented as the mean ± SEM of independent experiments and analysed by unpaired, two‐tailed *t*‐tests.

## CONFLICT OF INTEREST

The authors declare no conflict of interest.

5

### PEER REVIEW

The peer review history for this article is available at https://publons.com/publon/10.1111/tra.12831.

## Supporting information


**Figure S1** APP and BACE1 are segregated in the Golgi
A. Monolayers of HeLa cells stably expressing BACE1‐GFP were fixed, permeabilized and blocked and stained with anti‐APP(Y188) (red) and with anti‐EEA1 or anti‐Rab11 or anti‐CD63 (purple). Linescans were performed using Fiji. The colocalization coefficient (volume) of BACE1 and APP inside the different organelles staining was calculated using Imaris. Data are represented as the mean +/− SEM of three independent experiments (18 < n < 26).
B. Monolayers of HeLa WT cells were fixed, permeabilized. Blocked and stained with anti‐APPY188647 (purple) or with anti‐APPwo2 (green) or with anti‐APPY188 (green; LHS). Cells were also co‐stained with anti‐APPwo2 (green) and anti‐APP188647 (purple) or anti‐APPY188 (green) and anti‐APPY188647 (purple; RHS). Scale bars represent 10 μm.
C. Primary mouse cortical neurons were fixed at DIV7, blocked, permeabilized and co‐stained with different organelles markers: anti‐GM130 (mouse and rabbit), anti‐p230, anti‐GCC88, anti‐golgin97, anti‐LAMP1, anti‐CD63 (mouse). Colocalization was analysed using Imaris. The colocalization coefficient (volume) corresponds to the volume of the signal of marker A coincident with the signal volume of marker B (colocalization A and B) over the total signal volume of marker A. *cis* (GM130) versus *trans* (p230) colocalization coefficient is 0.19; *trans* (golgin97) versus *trans* (p230) colocalization coefficient is 0.49. GM130 (mouse antibody AF488) versus GM130 (rabbit antibody and AF562) colocalization coefficient is 0.97. Data are represented as the mean +/− SEM of a minimum of three independent experiments (26 < n < 42).
D. Primary mouse cortical neurons were fixed at DIV7, blocked, permeabilized and stained with anti‐APPY188647 (purple) or with anti‐BACE1(D10E5; green) and DAPI (blue). Scale bars represent 5 μm.
E. Primary mouse cortical neurons were fixed at DIV14, blocked, permeabilized and co‐stained with anti‐BACE1 (D10E5), anti‐APP(Y88647) and anti‐GM130 or anti‐golgin97 or anti‐p230. The colocalization coefficient (volume) of BACE1 and APP inside the Golgi were calculated using Imaris. Data are represented as the mean +/− SEM of four independent experiments (13 < n < 22).

**Figure S2** Volume of BACE1 and APP (voxels) in the Golgi
A. Volume (voxels) of the endogenous BACE1 and endogenous APP (HeLa WT) in GnT1‐GFP, Scarlet‐Giantin and p230 mask. Data is an extension of Figure 2A.
B. Volume (voxels) of the BACE1‐GFP and OKT9 (HeLa BACE1‐GFP) in GM130, golgin97, GCC88, GCC88 and golgin97 (TGN) and p230 mask. Data is an extension of Figure 2C.
C. Volume (voxels) of the endogenous BACE1 and endogenous APP in primary mouse neurons DIV 7 in GM130, golgin97 and p230 mask. Data is an extension of Figure 2F.

**Figure S3** The Golgi ribbon is not required for BACE1 and APP segregation
A. Monolayers of HeLa WT cells were treated with 10 μM nocodazole for 2 h at 37°C. Cells were fixed and permeabilized and stained with anti‐GM130, anti‐golgin97 and anti‐p230 or anti‐GM130, anti‐GCC88 and anti‐p230. Scale bars represent 5 μm or 1 μm (zoom), as indicated.
B‐D. Monolayers of HeLa cells stably expressing BACE1‐GFP were treated with 250 nM DAPT for 16 h and 10 μM nocodazole for 2 h at 37°C. Cells were fixed and permeabilized and stained with anti‐APP (red) and anti‐GM130 or anti‐golgin97 or anti‐golgin97 mixed with anti‐GCC88 (TGN; purple). Linescans were performed using Fiji. C. The colocalization coefficient (volume) of BACE1‐GFP and APP were calculated using Imaris. Data are represented as the mean +/− SEM of three independent experiments (20 < n < 29).
D. Co‐occurrence of BACE1‐GFP and APP‐ in Golgi ministacks. Data are represented as the mean of three independent experiments (20 < n < 29). Pie charts represent the percentage of the co‐occurrence of BACE1‐GFP and APP in individual Golgi ministacks for each different condition. Golgi ministacks can contain only APP (red), only BACE1 (green), APP and BACE1 (orange) or can be empty for BACE1 and APP (gray).
E. Monolayers of HeLa cells stably expressing BACE1‐GFP were treated with 2 μM ß‐secretase/BACE1 inhibitor C3 for 16 h and 10 μM nocodazole for 2 h at 37°C. Cells were fixed, permeabilized and stained with anti‐APP(Y188) and anti‐GM130 or anti‐golgin97 or anti‐golgin97 mixed with anti‐GCC88 (TGN). Co‐occurrence of BACE1‐GFP and APP in Golgi ministacks. Data are represented as the mean of three independent experiments (18 < n < 26). Pie charts represent the percentage of the co‐occurrence of BACE1‐GFP and APP in individual Golgi ministacks for each different condition. Golgi ministacks can contain only APP (red), only BACE1 (green), APP and BACE1 (orange) or can be empty for BACE1 and APP (gray).

## Data Availability

The datasets generated during and/or analysed during the current study are available from the corresponding author on reasonable request.
