# Supplementary figures and images for: Segregation of the membrane cargoes, BACE1 and amyloid precursor protein (APP) throughout the Golgi apparatus
Source: Traffic. 2022 Feb 13;23(3):158–73. doi: 10.1111/tra.12831 (PMC9303681; doi:10.1111/tra.12831)

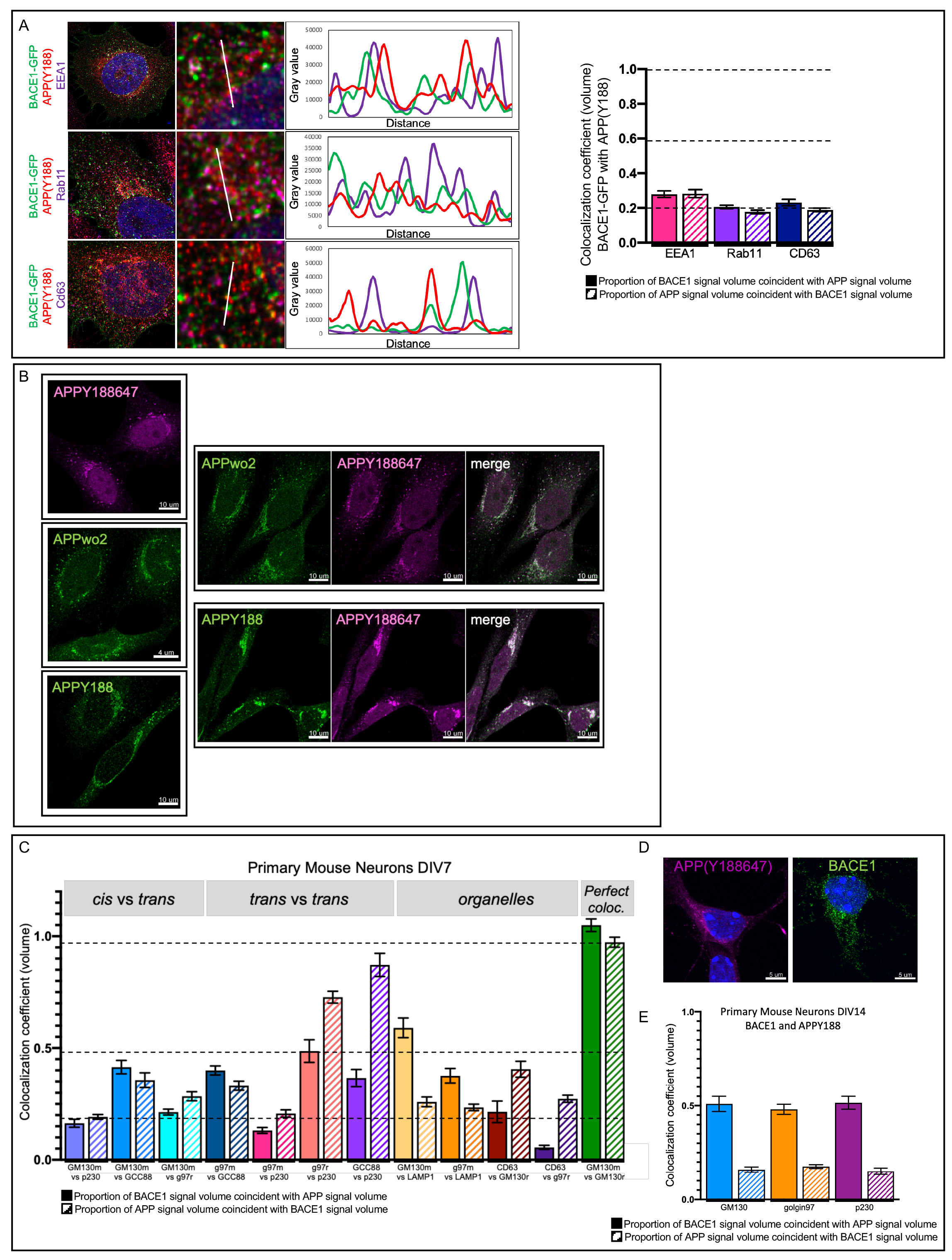

Supplement: Supplementary file 1 — Figure S1 APP and BACE1 are segregated in the Golgi A. Monolayers of HeLa cells stably expressing BACE1‐GFP were fixed, permeabilized and blocked and stained with anti‐APP(Y188) (red) and with anti‐EEA1 or anti‐Rab11 or anti‐CD63 (purple). Linescans were performed using Fiji. The colocalization coefficient (volume) of BACE1 and APP inside the different organelles staining was calculated using Imaris. Data are represented as the mean +/− SEM of three independent experiments (18 < n < 26). B. Monolayers of HeLa WT cells were fixed, permeabilized. Blocked and stained with anti‐APPY188647 (purple) or with anti‐APPwo2 (green) or with anti‐APPY188 (green; LHS). Cells were also co‐stained with anti‐APPwo2 (green) and anti‐APP188647 (purple) or anti‐APPY188 (green) and anti‐APPY188647 (purple; RHS). Scale bars represent 10 μm. C. Primary mouse cortical neurons were fixed at DIV7, blocked, permeabilized and co‐stained with different organelles markers: anti‐GM130 (mouse and rabbit), anti‐p230, anti‐GCC88, anti‐golgin97, anti‐LAMP1, anti‐CD63 (mouse). Colocalization was analysed using Imaris. The colocalization coefficient (volume) corresponds to the volume of the signal of marker A coincident with the signal volume of marker B (colocalization A and B) over the total signal volume of marker A. cis (GM130) versus trans (p230) colocalization coefficient is 0.19; trans (golgin97) versus trans (p230) colocalization coefficient is 0.49. GM130 (mouse antibody AF488) versus GM130 (rabbit antibody and AF562) colocalization coefficient is 0.97. Data are represented as the mean +/− SEM of a minimum of three independent experiments (26 < n < 42). D. Primary mouse cortical neurons were fixed at DIV7, blocked, permeabilized and stained with anti‐APPY188647 (purple) or with anti‐BACE1(D10E5; green) and DAPI (blue). Scale bars represent 5 μm. E. Primary mouse cortical neurons were fixed at DIV14, blocked, permeabilized and co‐stained with anti‐BACE1 (D10E5), anti‐APP(Y88647) and anti‐GM130 [file TRA-23-158-s001.tif]

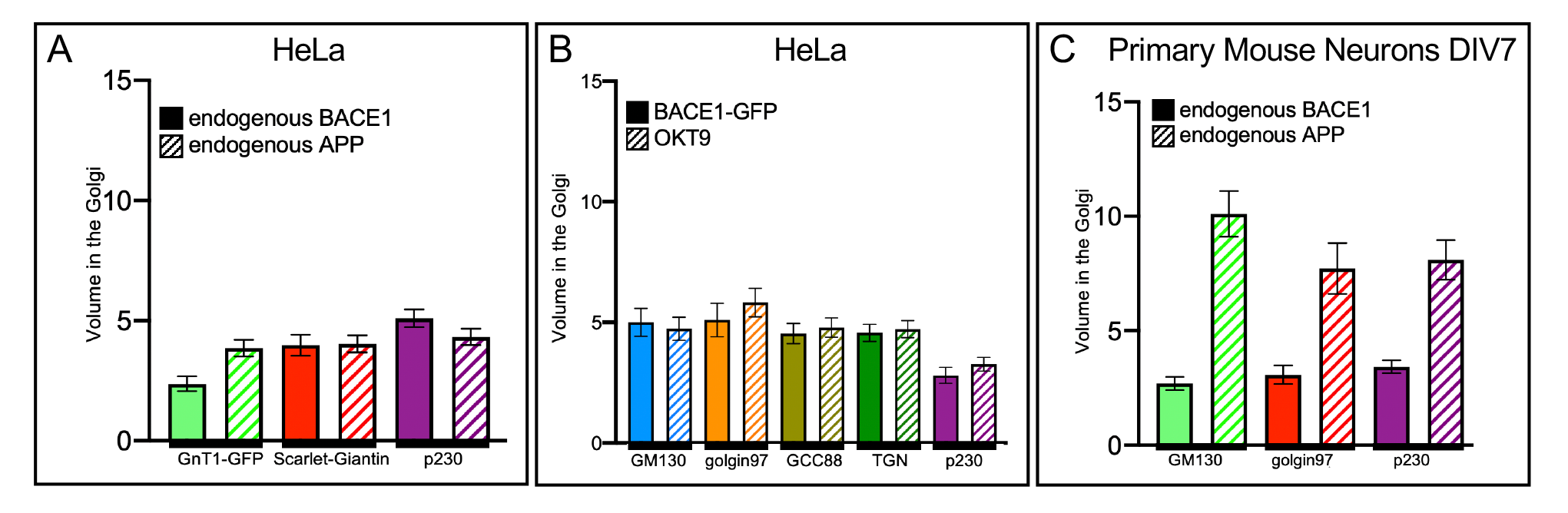

Supplement: Supplementary file 2 — Figure S2 Volume of BACE1 and APP (voxels) in the Golgi A. Volume (voxels) of the endogenous BACE1 and endogenous APP (HeLa WT) in GnT1‐GFP, Scarlet‐Giantin and p230 mask. Data is an extension of Figure 2A. B. Volume (voxels) of the BACE1‐GFP and OKT9 (HeLa BACE1‐GFP) in GM130, golgin97, GCC88, GCC88 and golgin97 (TGN) and p230 mask. Data is an extension of Figure 2C. C. Volume (voxels) of the endogenous BACE1 and endogenous APP in primary mouse neurons DIV 7 in GM130, golgin97 and p230 mask. Data is an extension of Figure 2F. [file TRA-23-158-s002.tif]

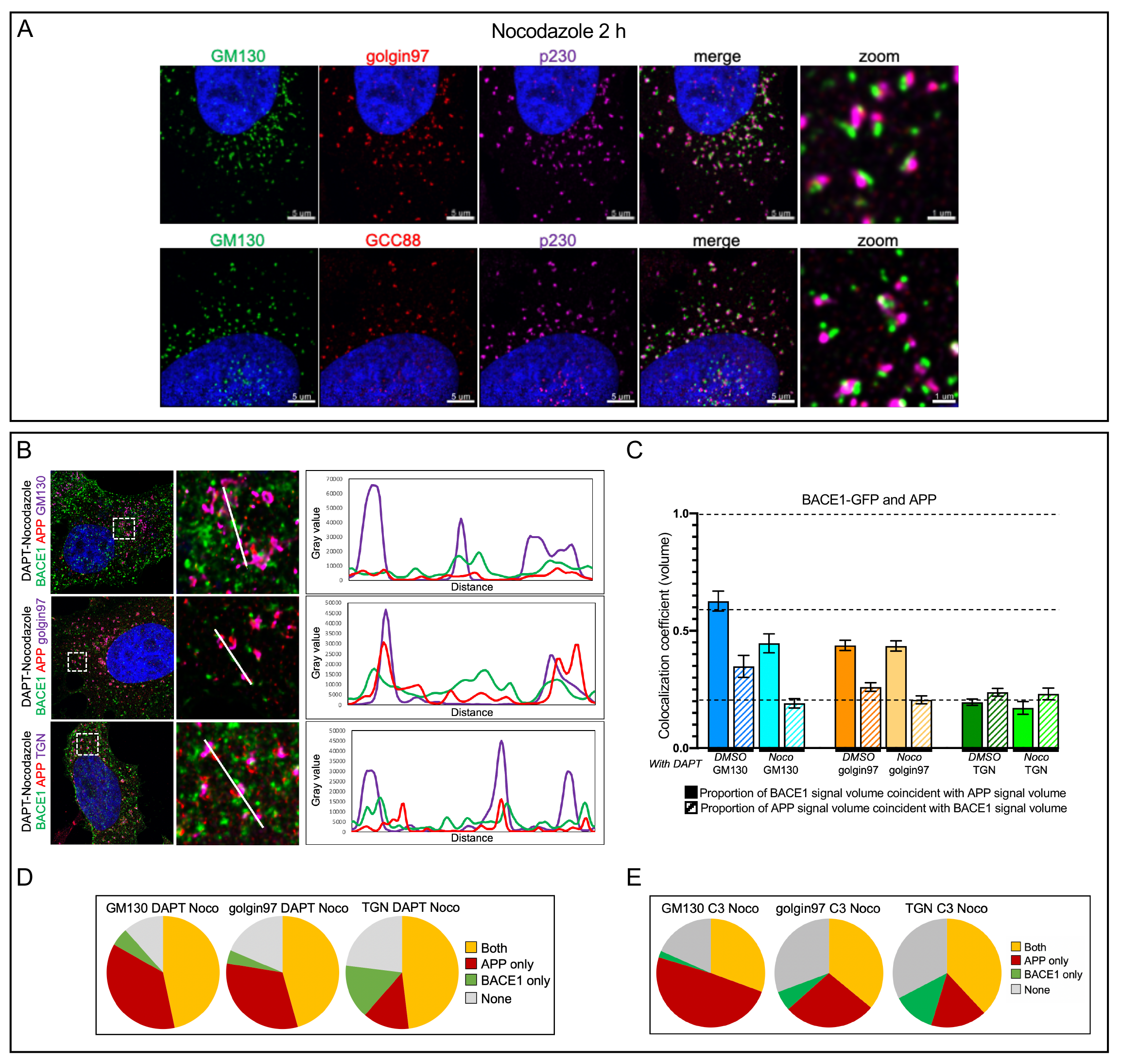

Supplement: Supplementary file 3 — Figure S3 The Golgi ribbon is not required for BACE1 and APP segregation A. Monolayers of HeLa WT cells were treated with 10 μM nocodazole for 2 h at 37°C. Cells were fixed and permeabilized and stained with anti‐GM130, anti‐golgin97 and anti‐p230 or anti‐GM130, anti‐GCC88 and anti‐p230. Scale bars represent 5 μm or 1 μm (zoom), as indicated. B‐D. Monolayers of HeLa cells stably expressing BACE1‐GFP were treated with 250 nM DAPT for 16 h and 10 μM nocodazole for 2 h at 37°C. Cells were fixed and permeabilized and stained with anti‐APP (red) and anti‐GM130 or anti‐golgin97 or anti‐golgin97 mixed with anti‐GCC88 (TGN; purple). Linescans were performed using Fiji. C. The colocalization coefficient (volume) of BACE1‐GFP and APP were calculated using Imaris. Data are represented as the mean +/− SEM of three independent experiments (20 < n < 29). D. Co‐occurrence of BACE1‐GFP and APP‐ in Golgi ministacks. Data are represented as the mean of three independent experiments (20 < n < 29). Pie charts represent the percentage of the co‐occurrence of BACE1‐GFP and APP in individual Golgi ministacks for each different condition. Golgi ministacks can contain only APP (red), only BACE1 (green), APP and BACE1 (orange) or can be empty for BACE1 and APP (gray). E. Monolayers of HeLa cells stably expressing BACE1‐GFP were treated with 2 μM ß‐secretase/BACE1 inhibitor C3 for 16 h and 10 μM nocodazole for 2 h at 37°C. Cells were fixed, permeabilized and stained with anti‐APP(Y188) and anti‐GM130 or anti‐golgin97 or anti‐golgin97 mixed with anti‐GCC88 (TGN). Co‐occurrence of BACE1‐GFP and APP in Golgi ministacks. Data are represented as the mean of three independent experiments (18 < n < 26). Pie charts represent the percentage of the co‐occurrence of BACE1‐GFP and APP in individual Golgi ministacks for each different condition. Golgi ministacks can contain only APP (red), only BACE1 (green), APP and BACE1 (orange) or can be empty for BACE1 and APP (gray). [file TRA-23-158-s003.tif]
